# ScRNA‐seq revealed disruption in CD8^+^ NKG2A^+^ natural killer T cells in patients after liver transplantation and immunosuppressive therapy

**DOI:** 10.1002/iid3.990

**Published:** 2023-09-27

**Authors:** Yuan Fang, CongWen Bian, ZhiTao Li, Li Jin, ChuHong Chen, YingLei Miao, HanFei Huang, Zhong Zeng

**Affiliations:** ^1^ Organ Transplantation Center the First Affiliated Hospital of Kunming Medical University Kunming Yunnan PR China; ^2^ Yunnan Province Clinical Research Center for Digestive Diseases Yunnan PR China

**Keywords:** immune tolerance, immunosuppressive therapy, KLRC1, liver transplantation, single‐cell RNA‐sequencing

## Abstract

**Background:**

Liver transplantation (LT) offers a good survival chance for both the patient in short or long term, but still faces many challenges in the treatment of LT, such as the side effects associated with long‐term immunosuppression, which is one of the side effects that occurs in most patients. However, the dynamics of the cellular immune system composition over time during immune tolerance to LT after immunosuppressive therapy are not known.

**Methods:**

Using single‐cell transcriptome sequencing, we analyzed five peripheral blood samples (one normal individual and four patients who underwent LT and received immunosuppressive therapy for 2 months, 1 year, 3 years, and 7 years, respectively) for immune cell composition and gene expression.

**Results:**

A total of 17,462 peripheral blood mononuclear cells were acquired from a normal individual without LT and patients who underwent LT and received immunosuppressive therapy for 2 months, 1 year, 3 years, and 7 years, respectively. A total of 24 cell clusters were obtained and categorized into four different cell types based on gene expression characteristics as follows: eight clusters of T cells, two clusters of B cells, two clusters of neutrophils, two clusters of monocytes, natural killer cells, and natural killer T (NKT) cells (*n* = 4), and six other cell clusters. Cell subset analysis, pseudotime analysis, and intercellular communication analysis revealed that the CD8^+^ NKT cells specifically expressed *NKG2A (KLRC1, CD159A)*, which may be an important cell group for CD8^+^ NKG2A^+^ NKT cells in LT, thereby highlighting the heterogeneity and functional diversity in patients who undergo LT.

**Conclusions:**

We comprehensively analyzed single‐cell RNA sequencing data from a normal individual and patients who underwent LT and elucidated the mechanism underlying the development of immune tolerance in LT. CD8^+^ NKT cells specifically expressing KLRC1 play a crucial role in LT, and dynamic monitoring of these cells may provide novel avenues for the diagnosis and treatment of LT‐related immune rejection.

## Graphical abstract

our study investigated the diversity and dynamic features of peripheral blood mononuclear cells in liver transplantation (LT) patients who were undergoing long‐term immunosuppressive therapy. Our findings shed light on the possible mechanisms of cellular interactions that contribute to immune tolerance in LT and provide insights for the development of more effective therapeutic targets and biomarkers for postoperative treatment.

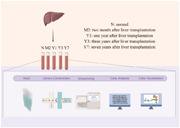

## 1 INTRODUCTION

Liver transplantation (LT) is a crucial surgical procedure that improves the quality of life for patients with acute or chronic liver disease, liver tumors, or certain inherited metabolic disorders. Research has shown that LT yields favorable short‐term and long‐term survival outcomes for patients.^1^ As an immunologically active organ, the liver interacts with the host and modulates the host's immune response, distinguishing it from other organs that can be transplanted. In fact, individual stable long‐term liver receptors may effectively counter the effects of immunosuppressive therapy without rejecting the graft.^2^ Nevertheless, compared to recipients of other organs, long‐term liver recipients encounter several daunting challenges, such as the side effects of immunosuppressive therapy, late graft failure, and chronic allograft injury. In the absence of immunosuppressive therapy, the recipient's mature immune system does mount an immune response against the graft, making donor‐specific immune tolerance the most favorable approach to post‐transplant immune tolerance for LT.^3^ A few studies suggest that certain patients can attain a state of immune tolerance after LT.^3^, ^4^ The liver possesses numerous intrahepatic innate immune cells, such as Kupffer cells, hepatogenic natural killer (NK) cells, and natural killer T (NKT) cells, and the immune responses in the liver are in a dynamic balance between tolerance and rejection by these cells, which distinguishes the liver from other solid organs.^5^ These cells play a critical role in the development of immune tolerance owing to their specific structure and function. For instance, NKT cells are primarily found in the liver, spleen, and bone marrow, which may express the molecular markers of NK cells.^6^ We hypothesize that during immunosuppressive therapy and the gradual development of immune tolerance in the recipient after LT, the recipient's immune system exhibits dynamic changes in the population distribution characteristics and gene expression patterns of immune cells to adapt to LT. Additional investigations of the molecular mechanisms underlying immune tolerance induced by innate immune cells after LT may provide insights into the tolerance function of the liver. In addition, the dynamics of the peripheral blood immune system at different time points after LT should be elucidated.

Single‐cell RNA sequencing (scRNA‐seq) is a technique that enables the amplification and sequencing of the entire transcriptome of individual cells, thereby providing high‐resolution gene expression profiles at the single‐cell level.^7^ Furthermore, this technique enables the determination of gene expression levels in individual cells, thereby accurately indicating the diversity and variation among immune cells. Additionally, it can be used to construct interaction networks between different cell populations.^8^ As a result, it has become a widely used approach to exploring gene expression in various cell types and determining the functions and mechanisms of different cells in these processes.^9^ To date, researchers have used single‐cell techniques to explore critical cell types and their gene functions in LT injuries caused by ischemia‐reperfusion.^10^ In addition, a preprint of an unpublished manuscript has reported the single‐cell profile of early dysfunction after LT and a pathogenic molecular module.^11^ However, the heterogeneity and dynamic changes of peripheral blood mononuclear cells (PBMCs) during immune tolerance to LT and the potential mechanisms of interactions between different cell subpopulations and their functions remain unclear in the medical field.

This is the first study to utilize scRNA‐seq to investigate lineage heterogeneity and functional status of distinct immune cell subpopulations and elucidate the dynamics of the immune system from 2 months to 7 years after LT. No previous study has utilized scRNAseq to examine the function and potential cellular and molecular mechanisms of different immune cell subpopulations during long‐term use of immunosuppressive drugs after LT. The findings of this study will serve as a foundation for further investigations into the functions and potential cellular and molecular mechanisms of different immune cell subpopulations during long‐term immunosuppressive therapy after LT.

## 2 METHOD

### 2.1 Collection of samples and scRNA‐seq

ScRNA‐seq data from five samples, that is, a normal individual (N) and four patients who underwent LT and received immunosuppressive therapy for 2 months (M2), 1 year (Y1), 3 years (Y3), and 7 years (Y7), respectively, were subjected to sequencing analysis to examine the changes in the immune system and identify specific key regulatory cell clusters and factors in the peripheral blood. M2 was in the early postoperative period following LT, having successfully overcome the stages of hyperacute rejection and acute rejection; Y1 was in the early postoperative period; Y3 was in the medium‐term postoperative period; and Y7 was in the long‐term postoperative period.^12^ Clinical information of all individuals is presented in Supporting Information: Table 1. ScRNA‐seq libraries were generated using Chromium Next GEM Single Cell 3ʹ Reagent Kits v3.1 (Dual Index) (×10 Genomics) and effectively dissociated single‐cell suspensions following the manufacturer's instructions and sequenced using the Illumina Novaseq. 6000 System. This study was approved by the Ethics Committee of the First Affiliated Hospital of Kunming Medical University.

### 2.2 Data preprocessing and quality control

The scRNA‐seq data of all five PBMC samples was converted into a Seurat object using Seurat in R (v4.0.4).^13^ Cells with <1000 UMIs, <500 detected genes, or >15% mitochondrial‐derived UMIs were considered low‐quality cells and excluded from analysis. Additionally, genes that were detected in <5 cells were excluded. We conducted principal component analysis of the integrated data matrix for dimensionality reduction of the data. The major cell clusters were identified using the “FindClusters” function of Seurat with default resolution (res = 0.6). Ultimately, cells were clustered into 24 distinct cell types and visualized via tSNE or uniform manifold approximation and projection (UMAP) plots. We used the “FindMarkers” function of Seurat to identify genetic markers and annotate cell types using previously reported immune system marker genes to identify each cell cluster type.^14^

### 2.3 Differentially expressed gene (DEG) analysis

Seurat was used to identify DEGs, employing a one‐tailed Wilcoxon rank sum test and Bonferroni correction for multiple testing. To further analyze DEGs, we examined all genes that exhibited an expression difference of at least 0.5 on a natural logarithmic scale and a difference of at least 0.15 in the percentage of cells they were detected in. Furthermore, we applied a significance threshold of an adjusted *p* value less than .05.

### 2.4 Monocle3 package was used to perform pseudotime trajectory analysis

The pseudotime trajectory of T cells was ascertained using Monole3 (v1.0.0).^15^ A standard workflow and default parameters were employed for dimensionality reduction and trajectory analysis.

### 2.5 Cell–cell communication

We analyzed cell–cell interactions and potential communication networks by inputting the normalized counts of known ligand–receptor pair expression in different cell types in “CellChat.” Preprocessing functions were used to set standard parameters for identifying overexpressed genes, upregulated interactions, and data projection.

### 2.6 Functional enrichment analysis

Functional categories of genes were identified using KOBAS 2.0 to ascertain Gene Ontology (GO) terms and KEGG pathways.^16^ The enrichment analysis for each term was performed using the hypergeometric test, and the false discovery rate was controlled by the Benjamini–Hochberg method.

### 2.7 Other statistical analysis

Euclidean distance‐based clustering was performed using the “pheatmap” package of R. For comparisons between two groups, *t*‐tests were employed.

## 3 RESULT

### 3.1 scRNA‐seq analysis of a normal individual and patients who underwent LT

**Figure 1:**
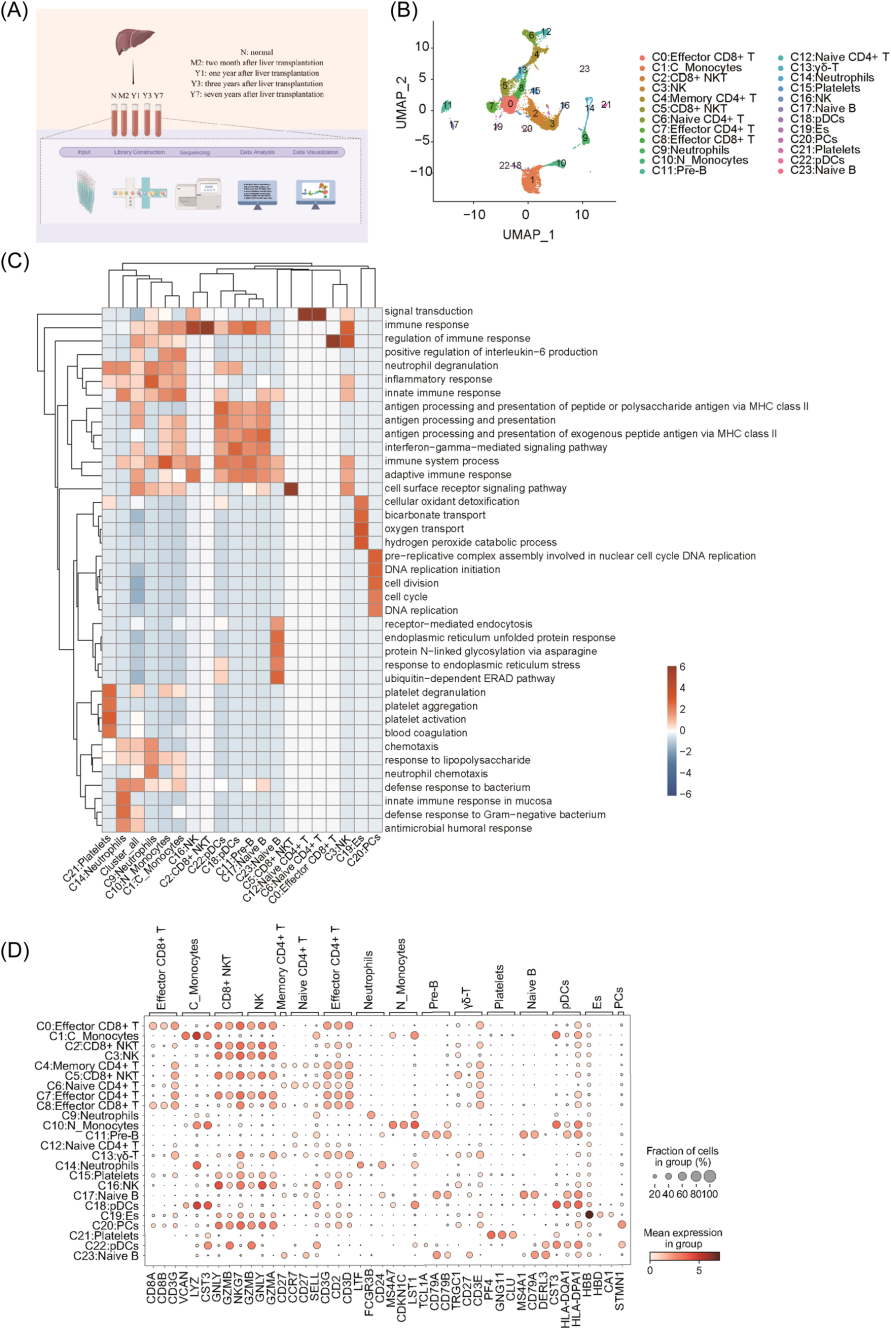
Global scRNA‐seq analysis of peripheral blood from a normal individual and patients who underwent LT revealed distinct cell clusters. (A) A schematic diagram of sample preparation and scRNA‐seq data processing workflow. (B) UMAP of integrated single‐cell transcriptomic profiles of all five samples, including a normal individual and patients who underwent LT. Colors indicate cell clusters along with annotations. (C) GO analysis was performed to identify biological processes associated with the top 50 marker genes within each cell cluster. The top three terms are based on enrichment q‐values within each cluster. The terms are presented in a heatmap. (D) A dot plot depicting the expression of selected genes in each cluster. Es, erythroid‐like and erythroid precursor cells; GO, Gene Ontology; PCs, progenitor cells; pDCs, plasmacytoid dendritic cells; scRNA‐seq, Single‐cell RNA sequencing.

ScRNA‐seq data from five samples, that is, those from a normal individual (N) and patients who underwent LT with immunosuppressive therapy for 2 months (M2), 1 year (Y1), 3 years (Y3), and 7 years (Y7), were analyzed to determine the changes in the immune system and identify specific key regulatory cell clusters and factors in the peripheral blood (Figure 1A). Following data quality control and filtering, we generated a transcriptome map of 17,462 single cells. Unbiased cluster analysis revealed 24 cell clusters. By examining DEGs within each cluster within each cluster and previously reported marker genes of peripheral hemocytes, we identified 16 distinct cell clusters (Figure 1B), including monocytes, neutrophils, B cells, T cells, and NK cells. The largest clusters were of T cells, NK cells, and progenitor cells, with erythroid‐like and erythroid precursor cells in the central region and situated before monocytes and plasmacytoid dendritic cells. Moreover, pre‐B and naïve B cells were observed on the left, and neutral granulocytes and a small fraction of platelets were observed on the right.

Next, we performed functional enrichment analysis of the top 100 marker genes in each cluster and found that each cell cluster exhibited specific enriched pathways, which were highly consistent with their cellular annotation results of clustering and functional enrichment analysis based on enriched *q* value (Figure 1C). Moreover, the expression of marker genes exhibited distinct characteristics (Figure 1D).

### 3.2 Single‐cell analysis revealed dynamic changes in cell clusters during immune tolerance after LT

**Figure 2:**
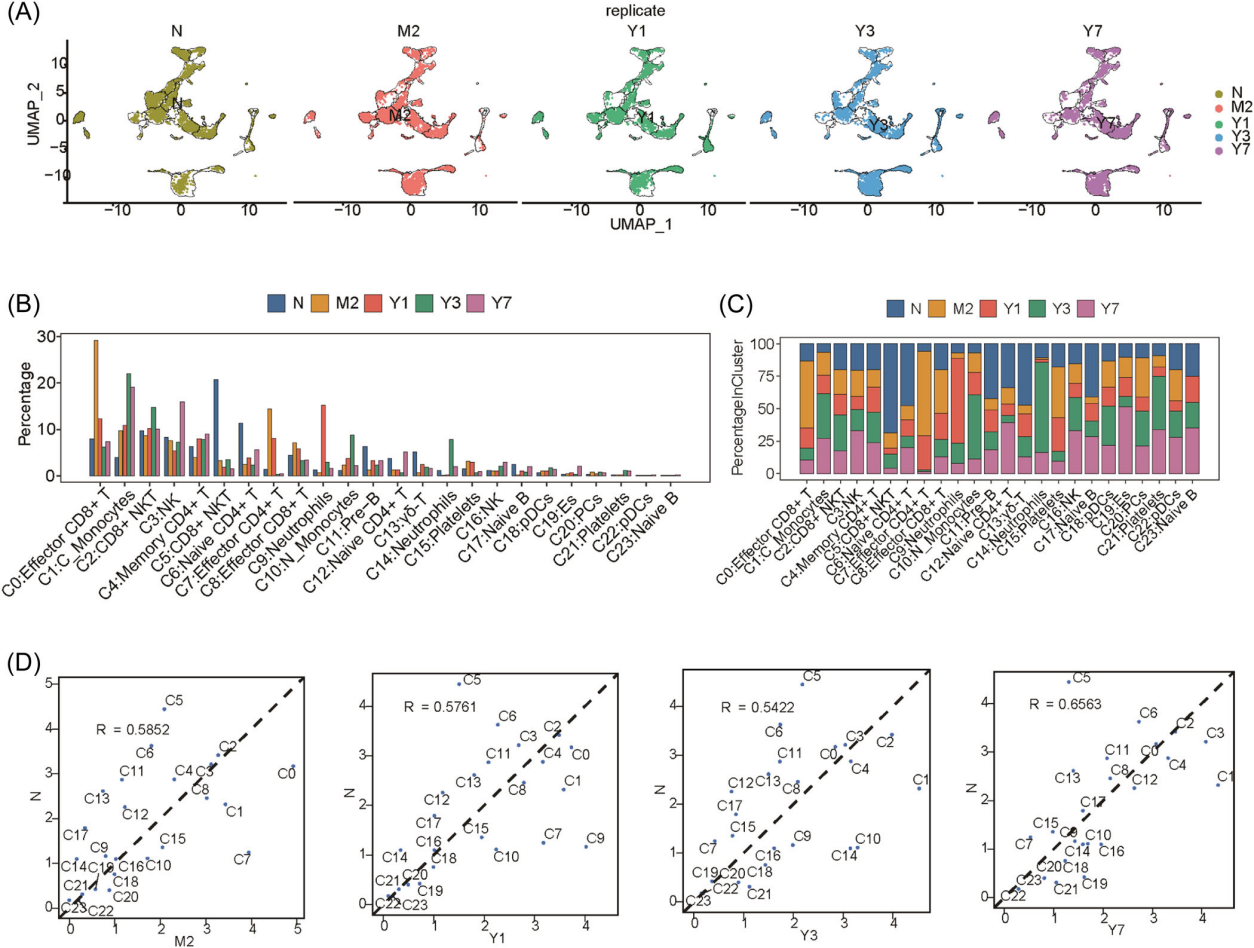
The dynamic changes of cell clusters in after LT. (A) UMAP plots of the different samples. (B) A bar plot showing the proportion of each cluster within each sample. (C) A stacked bar plot showing the relative proportion of each cell population. (D) A scatter plot showing the proportion of each cell cluster in the four samples. LT, liver transplantation.

To gain further insights into the effects of immunosuppressive therapy after LT, we analyzed the changes in hemocyte‐clusters during in vivo immune tolerance after LT. The hemocyte clusters exhibited more changes than N, highlighting the characteristics of the cell population and potential regulatory mechanisms (Figure 2A). Overall, compared to N, different cell clusters exhibited dynamic changes, with monocytes below, neutrophils on the right, and T and NK cells occupying the central region. Thus, we comprehensively analyzed the composition of each cell cluster in all five samples, which revealed notable changes in many cell clusters post‐LT (Figure 2B). In response to the changes in other cell clusters, the proportion of PBMC clusters could be notably changed after LT. Additionally, we analyzed the characteristic changes in the proportion of each cell cluster in all five samples and found that the proportion of 24 cell clusters exhibited dynamic changes (Figure 2C), consistent with the results shown in Figure 2B. Notably, C5, C6, C11, and C13 cell populations were remarkably decreased (*p* < .01), which accounted for more than 50% in N after LT. This may contribute to immune rejection in LT (Supporting Information: Figure S2). We compared the relevant characteristics of the cell populations between sample N and the four LT samples and found that although most of the cell clusters were close to the diagonal (insignificant changes in proportions), a few cell clusters exhibited notable deviations. Among them, C5 exhibited the most notable deviation. As shown in Figure 2A, a small number of C5 CD8^+^ NKT cells were present in the LT samples. Although a few cell populations were observed to be more prevalent in samples obtained from patients who underwent LT, this tendency was not consistent across the four samples obtained from patients who underwent LT, with C1 mononuclear cells showing relative consistency and supporting a higher proportion of cell populations in LT samples (Figure 2D).

### 3.3 Subcluster analysis revealed detailed immune cell heterogeneity in blood samples from patients who underwent LT

**Figure 3:**
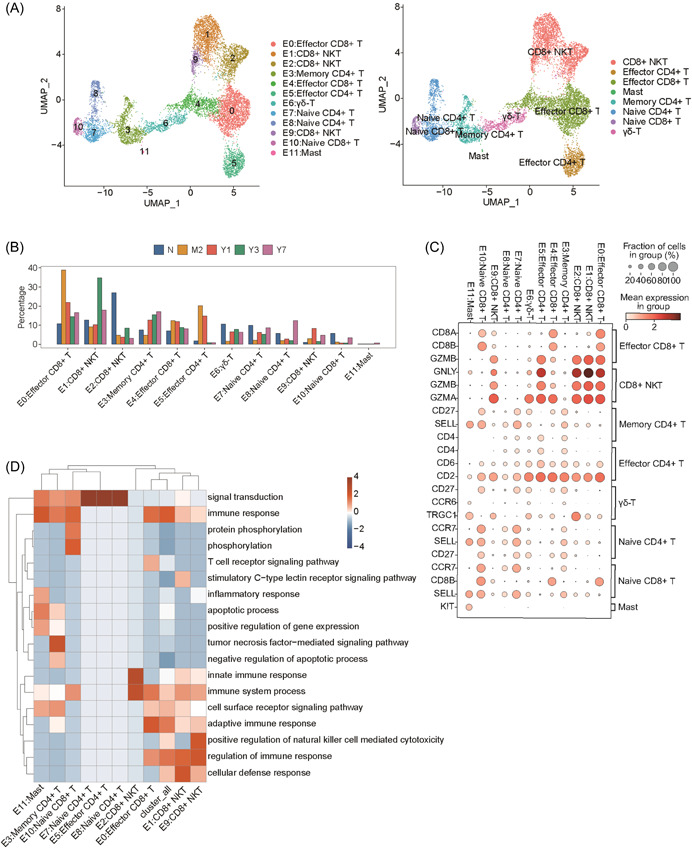
Subcluster analysis revealed immune cell heterogeneity. (A) The scRNA‐seq profile of T cells was used to generate a UMAP plot, which elucidated 11 cell subtypes. Cells are color‐coded based on their respective clusters. (B) A bar plot showing the proportion of T cells within each cluster. (C) A dot plot showing the expression of representative marker genes in each T cell cluster. (D) GO biological processes associated with the top 100 marker genes within each cluster. GO, Gene Ontology.

The aforementioned results suggested that the major cell clusters, that is, T and NKT cells, exhibited the most notable variation after LT. Hence, we focused on the variations of the T and NKT cell clusters, that is, the cells occupying the central region in Figure 1B. We identified 12 cell clusters by extracted and re‐UMAP clustered. After annotation, we identified eight different cell types (Figure 3A). We analyzed the proportions of these 12 cell clusters and found that they also exhibited dynamic changes. Among them, E2 CD8^+^ NKT cells were notably reduced after LT, consistent with the results pertaining to C5 in Figure 2. Furthermore, E1 CD8^+^ NKT cells were decreased in M3 and Y1 but were notably increased in Y3 and Y7 (Figure 3B). We speculated that these two types of CD8^+^ NKT cells may have different functions. We examined the marker genes of T and NK cells and found that they shared some common marker genes, including GzmB, GNLY, GzmA, and CD2. Notably, E2 CD8^+^ NKT cells exhibited a unique marker gene, namely TRGC1, which displayed notably low expression in other cell clusters (Figure 3C). As shown in Supporting Information: Figure S1, the other three marker genes (which showed the highest expression) in C5 CD8^+^ NKT cells were TRDC, TRGC1, and NKG2A (CD159A) (Supporting Information: Figure S1). Subsequently, we performed functional clustering analysis of the first 100 genes in each cell cluster and found that most of them exhibited pathways associated with immune response (Figure 3D).

### 3.4 CD8^+^ NKG2A^+^ NKT cells were dysregulated after LT and drug intaking

**Figure 4:**
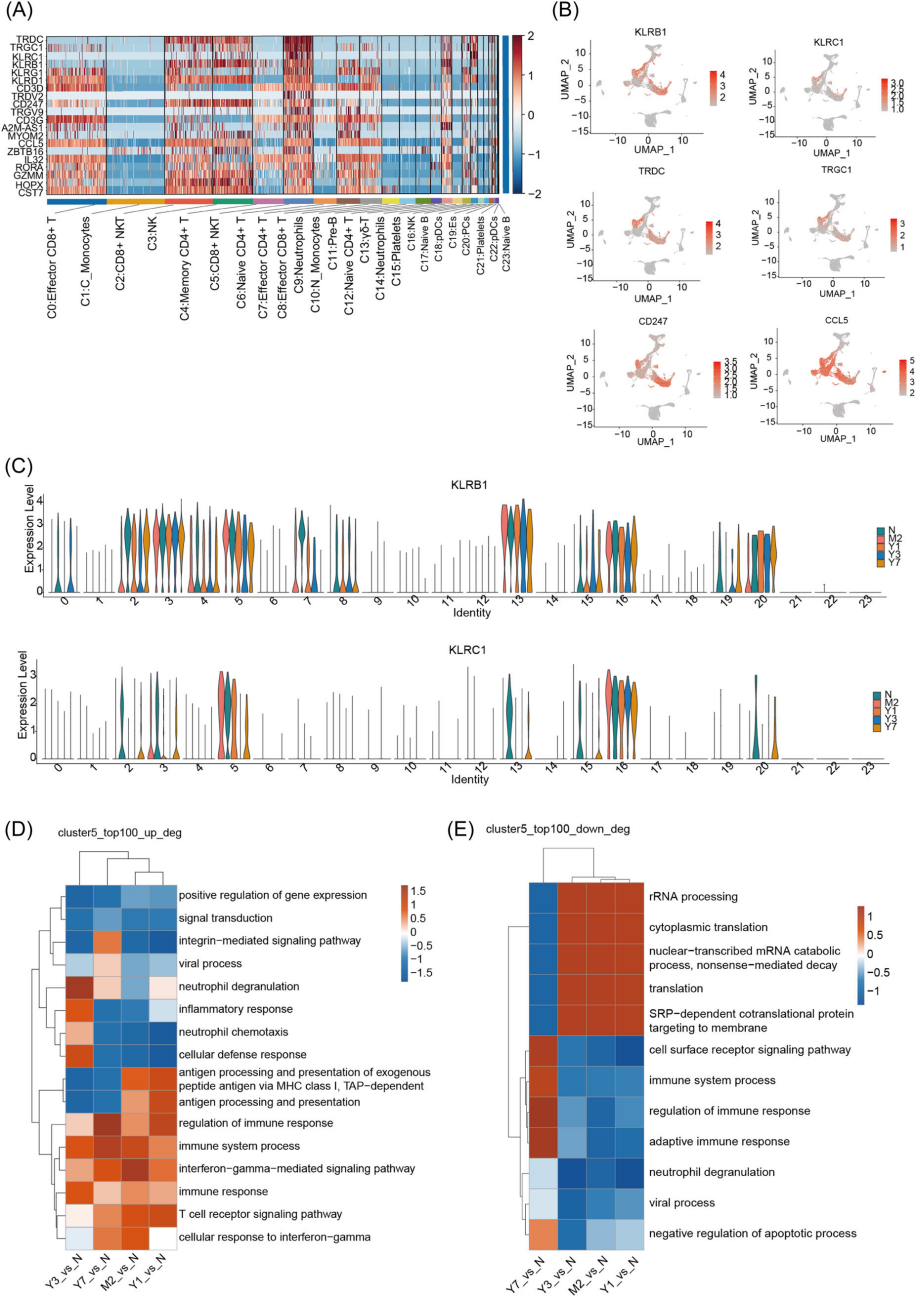
Dysregulation of CD8^+^ NKG2A^+^ NKT cells. (A) A heatmap showing the state of cluster‐specific regulons. Red indicates active, and blue indicates silenced. (B) UMAP plot of the expression of cluster 5 marker genes in all cell types. (C) A violin plot showing the expression levels of KLRB1 and KLRC1 in cluster 5. (D) The functional categories of the top 100 upregulated genes in cluster 5 were identified using GO biological process analysis of the four samples. (E) GO biological processes associated with the top 100 downregulated genes of cluster 5 in the four samples. GO, Gene Ontology; NKT, natural killer T.

Based on the aforementioned results, the major difference between N and M2, Y1, Y2, Y3, and Y7 was a group of CD8^+^ NKT cells, that is, C5, and E2 after T cell subdivision. C5 was the only cluster of CD8^+^ NKT cells, whereas C2, another cluster of CD8^+^ NKT cells, did not exhibit notable changes (Figure 2B). Consequently, we conjectured that the occurrence of immune rejection and immune tolerance during drug administration after LT could be attributed to C5 CD8^+^ NKT cells. We comprehensively analyzed the marker genes of C5 CD8^+^ NKT cells and found that the top‐ranked genes were TRDC, TRGC1, KLRC1, KLRB1, KLRG1, KLRD1, etc. (Figure 4A). We examined the expression characteristics of the most prominent genes, as well as the genes of CD247 and CCL5, in different cell clusters and found that they were expressed not only in C5 but also in a few other cell clusters, such as C2, another type of CD8^+^ NKT cells. Among them, KLRB1 (CD161) and NKG2A (CD159A) were highly expressed with good specificity in 24 cell clusters recovered expression (Figure 4B). Additionally, we observed that KLRB1 expression was also upregulated in other clusters except C5, and KLRC exhibited high specificity, primarily in C5 and C16 (Figure 4C). To determine cell specificity, we defined C5 and C16 as CD8^+^ NKG2A^+^ NKT cells and NK cells, respectively. To analyze the dynamic changes in C5 cell clusters during administration after LT, we performed differential gene expression analysis of C5 cell clusters and identified the DEGs in samples obtained from N, M2, Y1, Y3, and Y3. We identified the top 100 genes that exhibited the most notable differences and performed functional cluster analysis. The results showed that the upregulated genes in M2, Y1, Y3, and Y3 were enriched in pathways associated with the immune system, immune response, and inflammatory response (Figure 4D). The downregulated genes were principally enriched in pathways associated with rRNA processing, translation, and other related pathways, and those in Y7 Were only enriched in immune response‐related pathways (Figure 4E).

### 3.5 Dynamic changes in T and NK cells after LT

**Figure 5:**
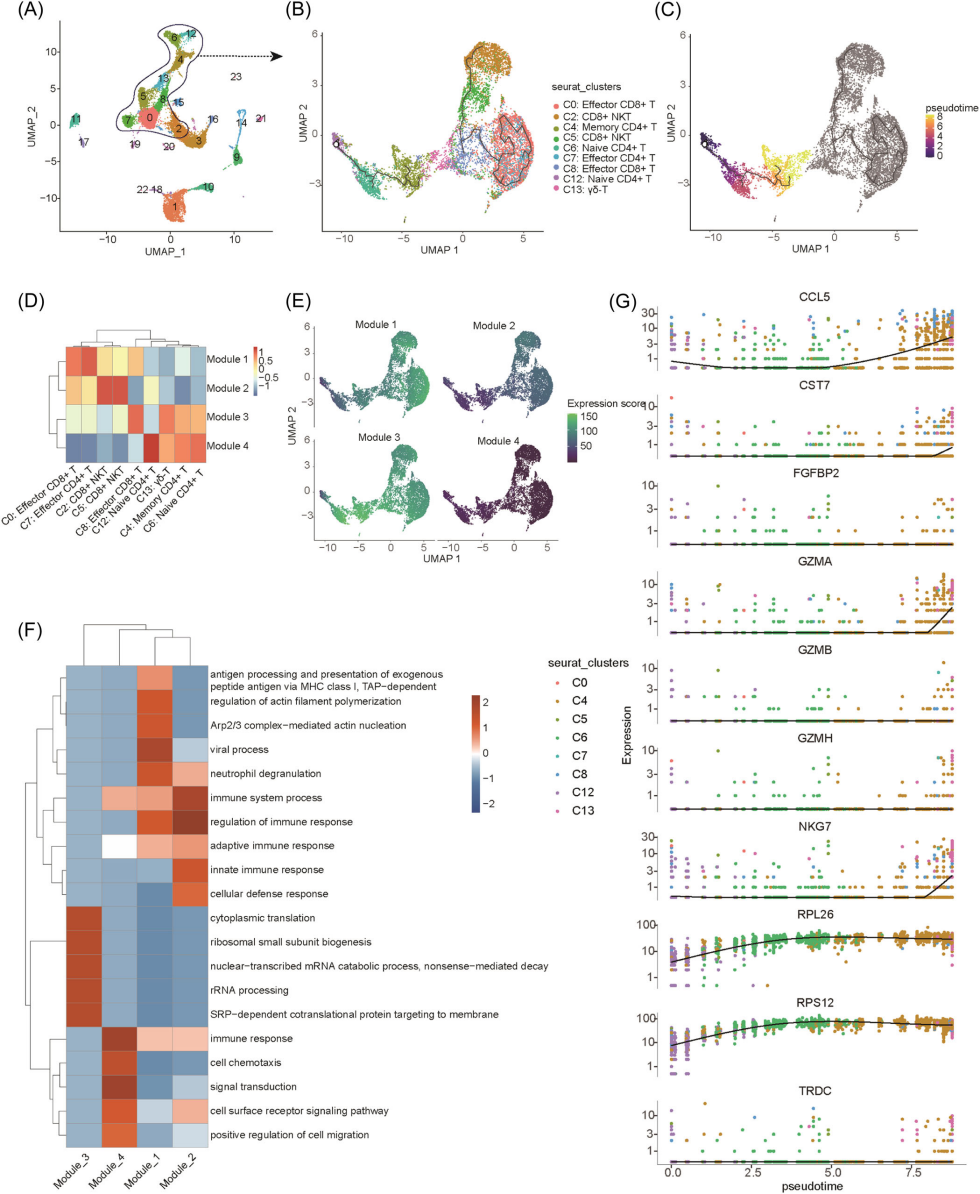
Dynamics of T and NK cells after LT. (A) T and NK cells were examined by pseudotime trajectory analysis. (B, C) Visualization of T cells arranged along trajectories using UMAP. Colors indicate the identified populations (B) or inferred pseudotime (C). (D) The clustering between different clustering modules and cell groups is shown in the form of a heatmap cluster diagram. (E) The expression of module genes in T and NK cells. (F) GO biological processes associated with genes in each module. (G) The variation in the expression of example cell markers along with pseudotime. GO, Gene Ontology.

To determine the characteristics and changes in T and NK cells after LT, we conducted a pseudotime trajectory analysis of these two types of cells and explored the potential impact of the changes and differentiation characteristics of immune cells on the immune system after LT (Figure 5A). We observed two relatively independent time axes. According to the results of the cell annotation, the time axis on the left represents the process of T cell differentiation and maturation, that is, from naïve CD8^+^ and CD4^+^ T cells to memory CD4^+^ T cells, γδ‐T cells, and ultimately to effector T cells, whereas the time axis on the right represents the relationship between effector T cells and NKT cells (Figure 5B). Together with the quasi‐sequential factor, it became evident that the time axis on the left exhibited a notable quasi‐sequential sequence. The time axis on the right was less obvious, gray, and consisted of primarily differentiated functional T and NK cells (Figure 5C). As shown in Figure 1, there were notable changes in the proportion of naïve CD4^+^ T cells (C6 and C12) before and after LT, which may lead to the disruption of T cell differentiation processes and ultimately affect the proportion and function of effector T cells. Based on the characteristics of the clusters, we further clustered the cell clusters using co‐expression methods and obtained a total of four expression modules. By performing association analysis between the four expression modules and nine cell groups, we determined the expression characteristics and clustering of the nine cell groups within the four expression modules. Modules 1 and 2 primarily consisted of effector T and NK cells, and modules 3 and 4 primarily consisted of memory T cells and naïve cells (Figure 5D). Overall, the scatter plot showed that modules 1 and 3 exhibited increased gene expression and modules 2 and 4 exhibited decreased gene expression (Figure 5E). To analyze the functional characteristics of the different expression modules, we conducted a functional clustering analysis of the highly expressed genes in each module. Regarding the enrichment pathways of each module, modules 1 and 2 were enriched in pathways associated with immune response; module 4 also exhibited similar characteristics; and module 3 was enriched in translation‐related pathways (Figure 5F). To summarize, through pseudo‐time trajectory analysis, we identified genes that exhibited varying expression and those that did not exhibit changes in their expression (Figure 5G).

### 3.6 Cell communication analysis revealed disruption of communication between CD8^+^ NTK cells and other cell modules after LT

**Figure 6:**
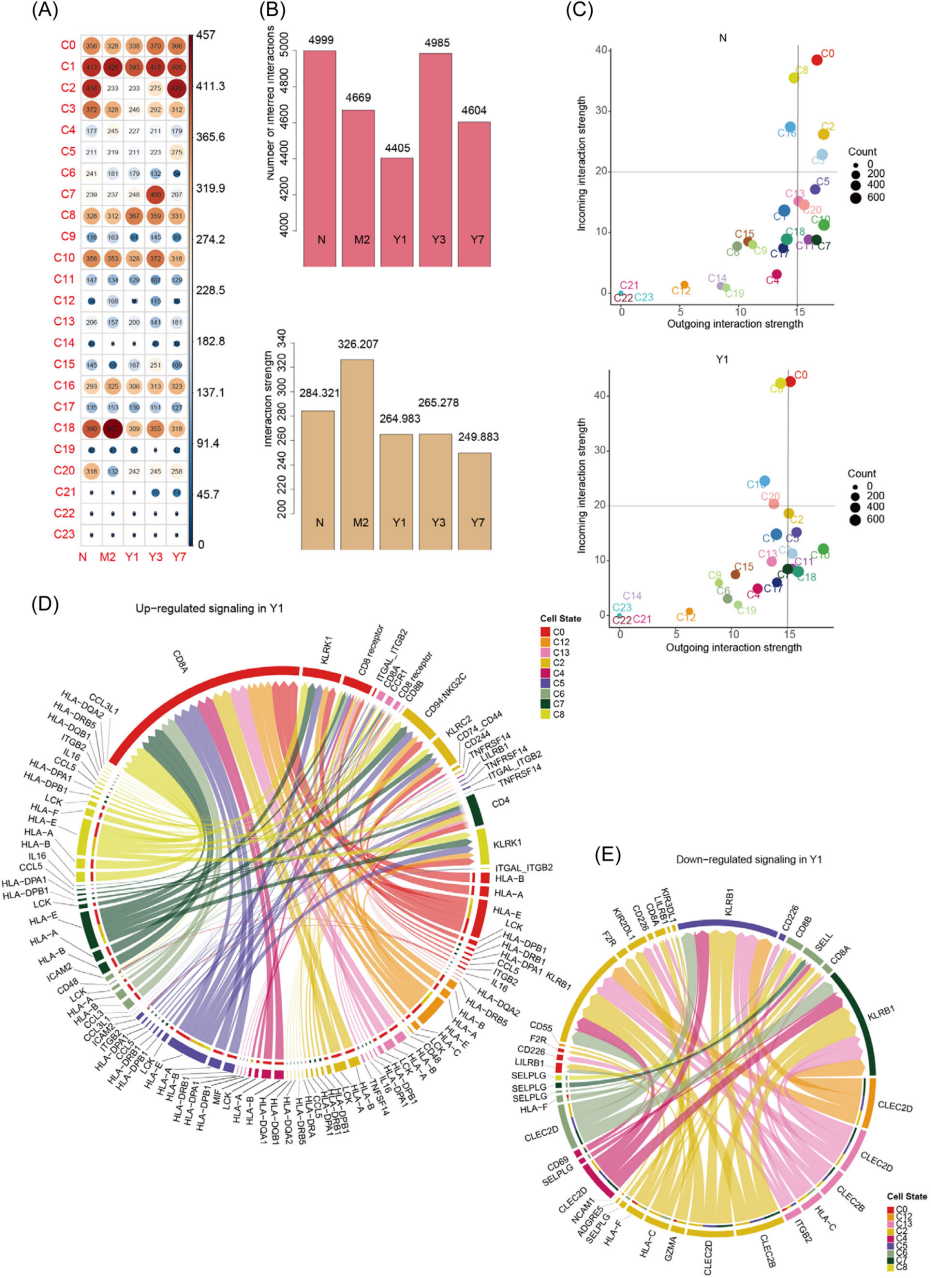
Disruption of CD8^+^ NTK cells after LT. (A) A dot plot showing the number of “CellChat”‐inferred interactions among each cell type in each sample. The color of the dots indicates the strength of interactions. (B) The frequency (up) and strength (down) of “CellChat”‐inferred interactions among cell populations in each sample. (C) Comparison of the strength of outgoing and incoming interactions in each cell type between a normal individual and a patient who underwent LT with 1 year of immunotherapy. (D, E) A circular plot displaying the upregulated and downregulated signals among nine cell clusters in Y1. LT, liver transplantation.

To elucidate the potential interactions between immune cell populations during immunosuppressive therapy after LT, we determined potential interactions among cell subpopulations in the different samples using “CellChat” and compared the changes in intercellular interactions during the advanced stage of immune rejection or tolerance after LT. We first examined the frequency and strength of cellular interactions for each cell population across the different samples. Compared to N, the four patients who underwent LT exhibited notable changes in the frequency and strength of cellular interactions among different cell populations, especially in the C2 cluster. For example, the C2 cluster exhibited notably decreased frequency and strength of intercellular communication in M2, Y1, and Y3, and later recovered in Y7 samples. The frequency and strength of cellular communication among C3 NK cells were also notably decreased and diminished after LT (Figure 6A). We also found that the frequency and strength of intercellular communication (except M2) in the four single‐cell sample groups were notably reduced, indicating that immunosuppressive therapy after LT possibly reduces the interactions among immune cells (Figure 6B). We compared the strength of efferent and afferent interactions among different cell groups in each sample. In Y1, the strength of afferent interactions among all cell populations, except C0 and C8, was decreased to varying degrees compared to that in N, and the strength of efferent interactions also showed similar changes, with C2, C3, and C5 exhibiting a more notable decrease. C5 represented a cluster of CD8^+^ NKT cells (Figure 6C). Similar results were observed for the changes in the strength of interactions in Y3 and Y7 (Supporting Information: Figure S3B,C). Through receptor ligand analysis, we found that the receptors CD8A and KLRK1 were notably upregulated in C0 and C8 in Y1 compared to N. Additionally, CD94 and KLRC2 in C2 and C7 and CD4 exhibited upregulated expression (Figure 6D). The signaling pathways in Y1, including KLRB1 in C2, C5, and C7, were upregulated, whereas in C2 and C6, they were downregulated (Figure 6E). In addition, analysis of up‐ and downregulated receptor–ligand pairs in Y3 and Y7 revealed similar results (Supporting Information: Figure S3D–F).

## 4 DISCUSSION

LT is commonly recommended for the treatment of end‐stage nonalcoholic liver disease, acute failure, metabolic disorders, or hepatocellular carcinoma. Currently, LT is a highly effective treatment for these indications.^17^ In patients who undergo LT with long‐term immunosuppressive therapy, peripheral blood cells are diverse, neutralize immune rejection/tolerance‐associated cells, and are essential for the regulation of immune tolerance after LT.^18^ In this study, we used scRNA‐seq to examine the heterogeneity and dynamic changes in peripheral blood cells in individual N and patients M2, Y1, Y3, and Y7 at the single cell level.

We identified 16 distinct PBMC clusters in patients who underwent LT with immunosuppressive therapy. This finding is consistent with the findings of a study that evaluated immune cells at the single‐cell level in the microenvironment of a steatotic liver graft in SD rats.^19^ This was our first striking finding. Furthermore, we found notable characteristic and functional differences in the expression profile and biological functions of different cell clusters, which were closely correlated with the degree of response and adaptation of the recipient's immune system after LT, thereby allowing the modulation of cell clusters by immunosuppressive therapy. M2 exhibited more interactions than the other clusters. Immunosuppressive therapy led to significant changes in the state and function of cells, resulting in changes in intercellular interactions, including changes in the intensity and number of interactions. Simultaneously, this change is dynamic over time, and there may be uncertainty. Eventually, the decrease in the frequency and intensity of cell–cell interactions may lead to homeostasis and equilibrium between the transplanted organ and the host.

Among the cell clusters, we observed many marker genes associated with allograft rejection, such as GNLY and GzmB in NKT and NK cells. GzmB is a unique serine protease that cleaves after aspartate. Furthermore, its activity is modulated by NK cells and activated T cell subsets.^20^ The utilization of GzmB in immunosuppressive therapy is an attractive idea given its efficiency in regulating apoptosis and immune tolerance as well as the recombinant and expressed function of humanized GzmB.^21^ Studies have shown that GzmB is associated with an early decline in lung function after lung transplantation and plays a crucial role in the process of acute rejection of lung transplantation.^22^ Recently, Tang et al. developed an intelligent near‐infrared fluorescent probe, namely CYGB, to visualize GzmB produced by cytotoxic T lymphocytes, thereby achieving early, noninvasive detection of allograft rejection.^23^

Analysis of cell subsets, quasi‐sequences, and intercellular communications revealed that Cluster 5 may play a critical role in immune tolerance to LT. Cluster 5 specifically expressed KLRC1 and may be a notable cluster of CD8^+^ NKG2A^+^ NKT cells in LT. These cells may be functionally dormant before LT but become activated after LT. However, the proportion of these cells was notably decreased and nearly diminished after drug administration. Therefore, we presume that they participate in immune rejection, and the decrease in their cell percentage following immunosuppressive therapy may be an important reason for their function. Additionally, an analysis of intercellular communication revealed that the frequency and intensity of intercellular communication were reduced under the influence of CD8^+^ NKG2A^+^ NKT cells, which exhibited upregulated expression of KLRC1 and even more upregulated expression of KLRB1. KLRB1‐expressing T cells exhibited varying degrees of NK cell‐like innate activity, which in some cases is considered a marker of “innate” T cells.^24^ NKT cells are a subgroup of lipid‐ and glycolipid‐reactive T lymphocytes that collectively express markers associated with NK cells. NKG2A, a member of the C‐type lectin‐like superfamily, is a surface receptor that is expressed as a heterodimer with CD94 in a subset of CD8^+^ T cells enriched in approximately 50% of NK cells and antigen‐experienced cells in human blood.^25^ Liu et al. found that blocking the interaction between HLA‐E and CD94^–^NKG2A restored the immune cytotoxicity mediated by NK cells in vivo and in vitro, thereby demonstrating the role of immune checkpoints.^26^ Hence, our results showed that CD8^+^ NKG2A^+^ NKT cells may be a cell population that is crucial to the development of immune tolerance to immunosuppressive therapy after LT and may be cells with immune rejection functions targeted by immunosuppressive therapy after LT, which is consistent with the findings of previous studies. Therefore, dynamic monitoring of CD8^+^ NKG2A^+^ NKT cells may enable the detection of the development of immune tolerance to immunosuppressive therapy after LT. Regarding the limitations of this study, we were unable to simulate clinical patients for validation in animal experiments. Additionally, more clinical samples are required to further validate our findings.

To summarize, we investigated the diversity and dynamic features of PBMCs in patients who underwent LT with long‐term immunosuppressive therapy. Our findings shed light on the possible mechanisms underlying cellular interactions that contribute to immune tolerance to LT and provide insights for the identification of more effective therapeutic targets and biomarkers for postoperative treatment.

## AUTHOR CONTRIBUTIONS

**HanFei Huang, YingLei Miao, and Zhong Zeng**: Conceptualization and financial support. **Yuan Fang and CongWen Bian**: Analyzed the data, carried out the main experiments, and drafted this manuscript. Contribution equals first author. All authors have read and agreed to the published version of the manuscript.

## CONFLICT OF INTEREST STATEMENT

The authors declare no conflict of interest.

## ETHICS STATEMENT

This study was approved by the Ethics Committee of the First Affiliated Hospital of Kunming Medical University.

## Supporting information

Supporting information.

Supporting information.

## Data Availability

The data used to support the findings of this study are available from the corresponding author upon request.
