# Supplementary material for: ScRNA‐seq revealed disruption in CD8+ NKG2A+ natural killer T cells in patients after liver transplantation and immunosuppressive therapy
Source: Immun Inflamm Dis. 2023 Sep 27;11(9):e990. doi: 10.1002/iid3.990 (PMC10524014; doi:10.1002/iid3.990)
Supplement: Supplementary file 1 — Supporting information. [file IID3-11-e990-s001.docx]

| **Sample** | **M2** | **Y1** | **Y3** | **Y7** |
| --- | --- | --- | --- | --- |
| **Age** | 54 | 57 | 58 | 56 |
| **Age at transplant** | 54 | 56 | 55 | 49 |
| **Native disease** | Non-alcoholic fatty liver disease | | | |
| **Type of transplant** | orthotopic liver transplantation | | | |
| **cold-ischemia time** | 3h | 2h | 3h | 3h |
| **warm-ischemia time** | 3min | 6min | 5min | 3min |
| **experience rejection episodes** | No | No | No | No |
| **clinical condition** | favorable outcomes | favorable outcomes | favorable outcomes | favorable outcomes |

Supplementary Table 1. Clinical information of all individuals.
