# Supplementary material for: ScRNA‐seq revealed disruption in CD8+ NKG2A+ natural killer T cells in patients after liver transplantation and immunosuppressive therapy
Source: Immun Inflamm Dis. 2023 Sep 27;11(9):e990. doi: 10.1002/iid3.990 (PMC10524014; doi:10.1002/iid3.990)
Supplement: Supplementary file 2 — Supporting information. [file IID3-11-e990-s002.docx]

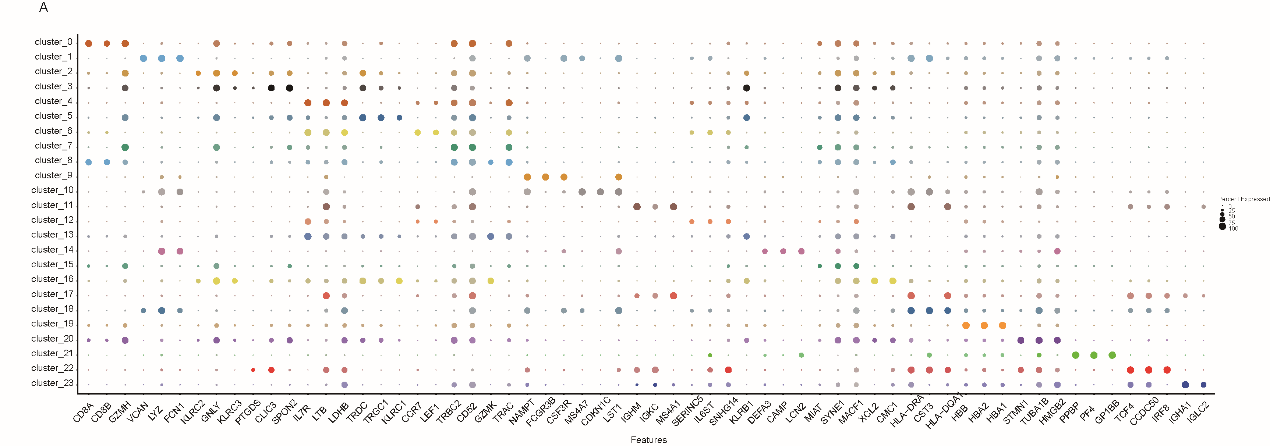


**Figure S1. Dot plot of top 3 marker genes of 24 cell clusters.**


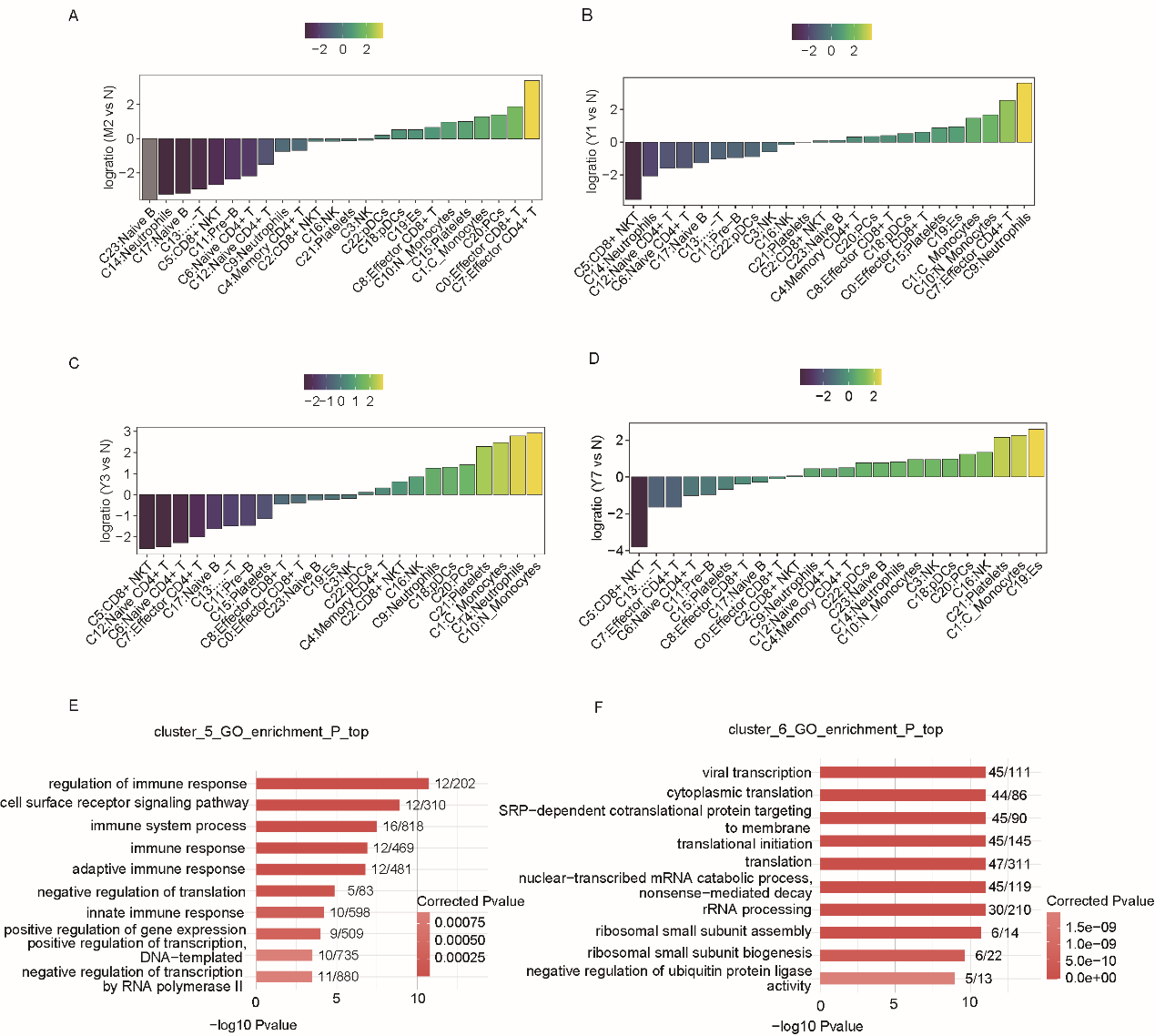


**Figure S2. ScRNA-seq analysis of PBMC from healthy donor and patients after liver transplantation identified distinct cells types.**

1. D) Rank order based on decreasing values of the relative frequency ratio among four sample groups.

F) Gene Ontology biological process of top 100 representative genes in cluster 5 (CD8+ NKT) and cluster 6 (Naive CD4+ T) cell type.
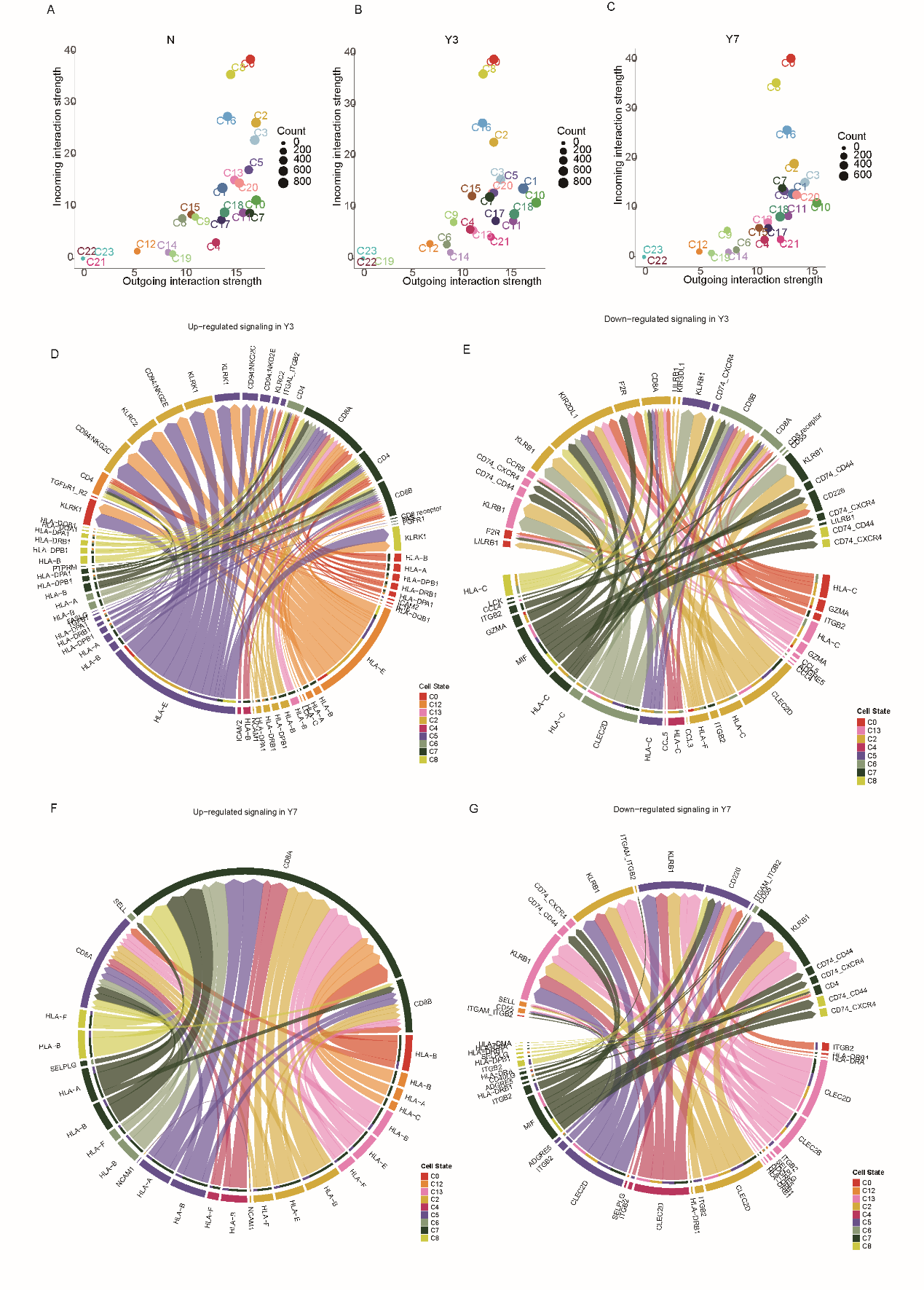


**Figure S3. Cell communication predicted by Cellchat**

(A-C) Comparing the outgoing and incoming interaction strength for each cell type between normal and liver transplantation samples Y3 and Y7.

1. G) Circular plot displays up-regulated and down-regulated ligand-receptor pairs of 9 cell clusters in sample Y3 and Y7. Lines originate at the ligand and connect to its receptor.
